# An Alternative Exploitation of *Synechocystis* sp. PCC6803: A Cascade Approach for the Recovery of High Added-Value Products

**DOI:** 10.3390/molecules28073144

**Published:** 2023-03-31

**Authors:** Paola Imbimbo, Luigi D’Elia, Iolanda Corrado, Gerardo Alvarez-Rivera, Antonio Marzocchella, Elena Ibáñez, Cinzia Pezzella, Filipe Branco dos Santos, Daria Maria Monti

**Affiliations:** 1 Department of Chemical Sciences, University of Naples Federico II, Via Cinthia 4, 80126 Naples, Italy; 2 Laboratory of Foodomics, Institute of Food Science Research, CIAL, CSIC, Nicolás Cabrera 9, 28049 Madrid, Spain; 3 Department of Chemical, Materials and Industrial Engineering, University of Naples Federico II, Piazzale Tecchio 80, 80125 Naples, Italy; 4 Molecular Microbial Physiology Group, Swammerdam Institute for Life Sciences, Faculty of Science, University of Amsterdam, Science Park 904, 1098 XH Amsterdam, The Netherlands

**Keywords:** cyanobacteria, biorefinery, high-value compounds, antioxidants, polyhydroxyalkanoate

## Abstract

Microalgal biomass represents a very interesting biological feedstock to be converted into several high-value products in a biorefinery approach. In this study, the cyanobacterium *Synechocystis* sp. PCC6803 was used to obtain different classes of molecules: proteins, carotenoids and lipids by using a cascade approach. In particular, the protein extract showed a selective cytotoxicity towards cancer cells, whereas carotenoids were found to be active as antioxidants both in vitro and on a cell-based model. Finally, for the first time, lipids were recovered from *Synechocystis* biomass as the last class of molecules and were successfully used as an alternative substrate for the production of polyhydroxyalkanoate (PHA) by the native PHA producer *Pseudomonas resinovorans*. Taken together, our results lead to a significant increase in the valorization of *Synechocystis* sp. PCC6803 biomass, thus allowing a possible offsetting of the process costs.

## 1. Introduction

**Figure 1:**
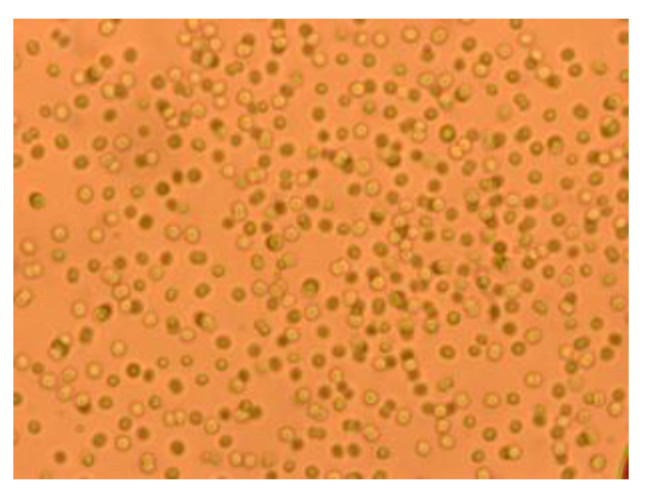
Optical microscopy image collected at 1000-fold magnification of a stably growing population of *Synechocystis* sp. PCC6803.

The dramatic overproduction of greenhouse gas emissions is due to many factors, among which is an increasing demand of essential goods, whose production still relies on fossil resources [1,2,3]. In the context of the circular bioeconomy, microalgae and cyanobacteria have emerged as an important feedstock, as these microorganisms produce molecules which can be used as renewable raw materials to substitute fossil-based fuels, chemicals, food and plastics [4,5,6]. In addition, microalgae and cyanobacteria require a much smaller land area to produce high amounts of biomass compared to conventional crops [7] and have a very high productivity [8]. Last but not least, microalgae fix CO_2_ during photosynthesis (about 1.8 kg of CO_2_ is fixed for each kg of microalgae biomass) [3,9]. Unfortunately, the extraction of molecules from microalgal biomass suffers from many limitations, among which the scaling up of lab to industry is one of the most important. Indeed, laboratory-scale transitions and industrial applications are hindered by a plethora of factors, comprising up- and down-stream processes, that can have a negative effect on the full exploitation of microalgae at industrial level. Therefore, to overcome these problems, and to emphasize profitable benefits, biorefinery approaches have to be proposed. Among cyanobacteria, *Synechocystis* sp. PCC6803 is a strain of unicellular freshwater microorganism, isolated from a freshwater lake in 1968, and mostly used as a source of lipids (Figure 1) [10]. Moreover, *Synechocystis* sp. PCC6803 can readily take up exogenous DNA [11], thus genetically engineered strains have become very common to enhance the production of a wide variety of compounds, such as poly-β-hydroxybutyrate [12,13] and ethylene [14,15]. Coupling biomass production with wastewater bioremediation enhanced the profitability of this cyanobacterial-based cell factory [16,17]. It has to be pointed out, however, that the proposed approach is intended to lower the upstream costs but does not exploit the biomass, which instead is a reservoir of high added-value molecules. To the best of our knowledge, the approach proposed in this paper is completely different and innovative, as the attention was focused not on the costs related to the cultivation strategy, but on the extraction of three different classes of molecules: proteins, carotenoids and lipids, all of them with a high market value, and with a wide range of applications in different fields. Moreover, the presented strategy completely fulfills the biorefinery approach, as the cascade extraction selected allows the obtainment of different classes of molecules without affecting the activity of the remaining molecules still present in the leftover biomass.

## 2. Results

### 2.1. Synechocystis sp. PCC6803 Cultivation

As described in the Materials and Methods section, *Synechocystis* sp. PCC6803 was grown autotrophically in bubble column photobioreactors (PBRs) in BG-11 medium at pH 6.5, at 30 ± 1 °C, with a constant light of 200 PAR (μmol_photons_ m*^−^*^2^ s*^−^*^1^]. The biomass productivity of *Synechocystis*, during the exponential growth phase, was 1.7 ± 0.1 g_D.W._ L*^−^*^1^, five and two times higher than those observed by Touloupakis (0.355 ± 0.02 g_D.W._ L*^−^*^1^) [18] and by Straka (0.760 g_D.W._ L*^−^*^1^) [19].

### 2.2. Biorefinery Strategy

**Figure 2:**
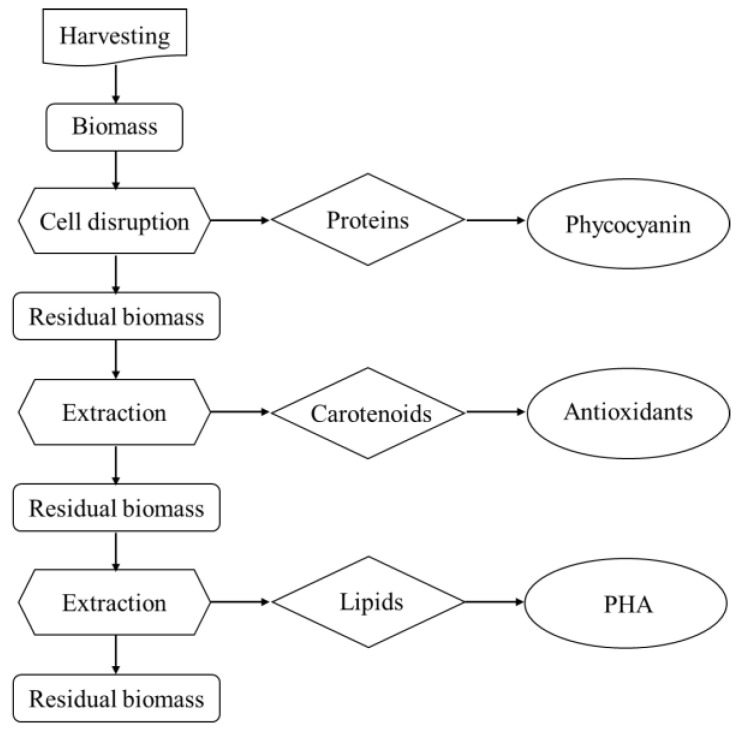
Schematic representation of the biorefinery strategy.

Figure 2 represents the strategy used for the biorefinery approach. In particular, the order of the extraction steps was dictated by the polarity of the class of molecules to be extracted, to ensure that the solvents used (water, ethanol, chloroform/methanol) would not affect the residual biomass quality. Since proteins were the class of molecules most susceptible to degradation, they were extracted first. Then, by increasing the hydrophobicity of the solvents, pigments and lipids were obtained as second and third class of molecules, respectively.

#### 2.2.1. Protein Extraction

Proteins were extracted from the wet biomass as described in the Materials and Methods section, and the yield was 233 ± 15 mg g_D.W._^−1^. These were analyzed by SDS-PAGE followed by Coomassie staining (Supplementary Materials, Figure S1). The analysis revealed the presence of two major protein species, whose molecular weight corresponded to those of α- and β-subunits of phycocyanin (PC, 17 and 18 kDa, respectively) [20]. PC extraction yield was 141 ± 1 mg _gD.W._^−1^ and the ratio PC/proteins was 49 ± 12%. The PC purity grade value was 1.8 ± 0.6 which corresponded to a reagent grade [21]. The ratio between total PC and total proteins, as well as the purity grade, were higher than those previously reported by Gorgich [22].

#### 2.2.2. Pigments Extraction and Identification

**Figure 3:**
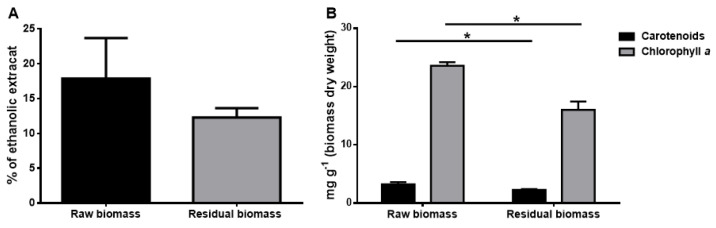
Carotenoids extraction from *Synechocystis* sp. PCC6803. (**A**) Yields are reported as % with respect to dry weight biomass. Black bar is referred to untreated biomass and grey bar is referred to residual biomass after protein extraction (*p* > 0.05). (**B**) Total carotenoids (black bars) and chlorophyll *a* (grey bars) extracted from raw and residual biomass. Each content is expressed as mg of extract pigment per g of biomass dry weight. * is referred to *p* < 0.05. The line is used to show which samples have been used to carry out statistical analysis.

According to the cascade approach, pigments were extracted as the second class of molecules. Ethanol, a GRAS solvent, was used to obtain pigments from the residual biomass, i.e., the biomass left after protein extraction. As shown in Figure 3A, the yield of the ethanolic extract after protein extraction (grey bar) was 12 ± 1%, about 70% of the yield obtained from the raw biomass (black bar, 18 ± 6%). As shown in Figure 3B, data indicated that carotenoid content (black bars) was not critically altered after protein extraction (2.2 ± 0.1 mg g_D.W._^−1^ in the residual biomass vs. 2.8 ± 0.5 mg g_D.W._^−1^ in the raw biomass) and, interestingly, a 30% decrease in chlorophyll *a* level was observed (15.8 ± 1.5 mg g_D.W._^−1^ and 24.0 ± 1.2 mg g_D.W._^−1^, respectively) (grey bars). This result is quite interesting, as, after protein extraction, the extract showed a lower amount of chlorophyll *a*, which is believed to reduce the quality of the pigments’ extracts [23]. Extracts were also analyzed by HPLC, which revealed that zeaxanthin and β-carotene were the major pigments present in the two extracts (Supplementary Materials, Figure S2 and Table S1). Quantification analyses were done to determine the amount of carotenoids, and, as reported in Table 1, the amount of zeaxanthin present in the residual biomass represented about 60% of that found in the raw biomass, whereas β-carotene isoforms recovery were about 80% with respect to the raw biomass.

#### 2.2.3. Lipidic Fraction Extraction and Quantification

**Figure 4:**
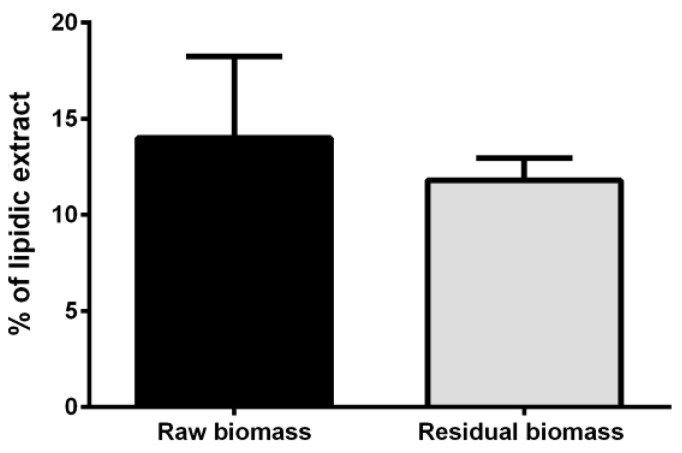
Lipidic extract from *Synechocystis* sp. PCC6803. Yields are reported as % with respect to dry weight biomass. Black bar is referred to untreated biomass and grey bar is referred to residual biomass post proteins and carotenoids extraction.

Lipids are an important class of molecules in cyanobacteria, as they are normally present at significant levels (15–20% of dry weight biomass) [24,25,26]. For this reason, lipids were considered in the biorefinery strategy and were extracted as the last class of molecules. In particular, the lipidic fraction was obtained from the residual biomass by a conventional method previously described [27]. To this purpose, the residual biomass after protein and pigments extraction was dried, weighed and further used. In a parallel experiment, a lipidic extraction was performed on the raw biomass. As shown in Figure 4, the hydrophobic fraction obtained from the residual biomass was 12 ± 1%, a value similar to that obtained from the raw one (14 ± 4%), thus suggesting that previous extractions do not significantly alter lipids yield (*p* > 0.05).

### 2.3. Biotechnological Application of the Extracted Molecules

#### 2.3.1. Protein Fraction

**Figure 5:**
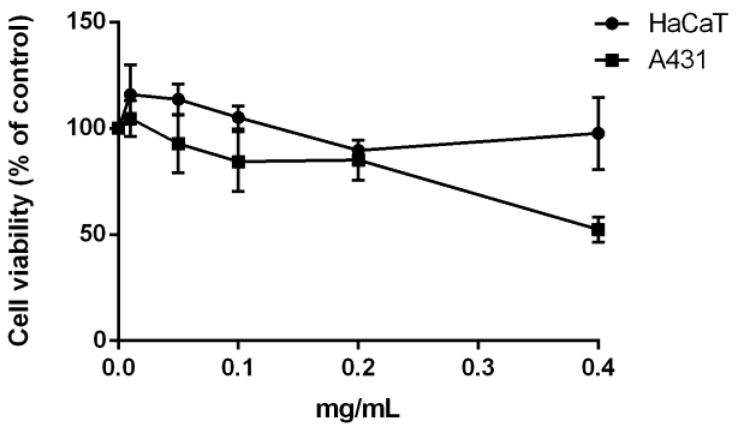
Effect of the protein extract on cell viability. Dose-response curve of HaCaT cells (black circles) and A431 cells (black squares) incubated for 48 h in the presence of increasing concentration (0.01–0.4 mg mL^−1^) of protein extract. Cell viability was assessed by the MTT assay and expressed as described in the Materials and Methods section. Values are given as means ± SD (*n* ≥ 3).

The biological effect of the aqueous extract containing proteins was evaluated on the viability of eukaryotic cells. Thus, the extract obtained from the fresh biomass was tested on human cell lines. Immortalized human keratinocytes (HaCaT) and human epidermoid carcinoma cells (A431) were treated with increasing amount of extract (from 0.01 to 0.4 mg mL^−1^). After 48 h incubation, cell viability was assessed by the MTT assay (3-(4,5-dimethylthiazol-2-yl)-2,5-diphenyltetrazolium bromide). Cell viability was expressed as the percentage of viable cells in the presence of the aqueous extract compared to that of control (i.e., untreated cells and cells incubated with the buffer, as described in the Materials and Methods section). As shown in Figure 5, the protein extract was found to be fully biocompatible on immortalized cells (HaCaT), whereas it exerted a selective effect on cancer cells, as a 50% reduction in cell viability was observed (IC_50_ on A431 cells of 0.39 ± 0.01 mg mL^−1^). It is known that phycocyanin (PC) extracted from other microalgal strains is endowed with a selective toxic effect towards cancer cells [28]. As in this experimental system the protein extract is enriched in PC (about 50%), it is possible to speculate that PC is the protein responsible for the selective toxicity observed.

#### 2.3.2. Carotenoids

**Figure 6:**
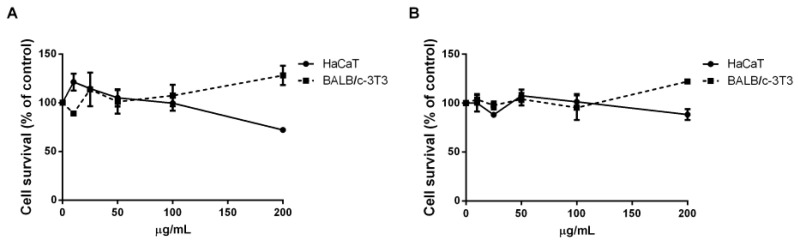
Effect of ethanolic extracts from *Synechocystis* sp. PCC6803 on the viability of immortalized cells. Dose-response curve of cells incubated for 48 h in the presence of increasing concentration (10–200 μg mL^−1^) of each extract. Cells were incubated with ethanol extracts from both raw (**A**) and residual (**B**) biomass on HaCaT (circles) and BALB/c-3T3 (squares). Cell viability was assessed by the MTT assay and expressed as percentage of the control. Values are given as means ± SD (*n* ≥ 3).

As carotenoids are more and more often sold for cosmeceutical and nutraceutical applications, it is mandatory to verify their effect on cell viability. To this purpose, their biocompatibility was verified before studying their antioxidant activity in skin protection. Thus, a MTT assay on eukaryotic immortalized skin cell lines was performed. In particular, HaCaT (human keratinocytes) and BALB/c-3T3 (murine fibroblasts) were tested as they mimic the outermost skin layer and the dermis, respectively. Cells were incubated for 48 h with increasing concentrations of the extracts (from 10 to 200 µg mL^−1^) obtained from untreated and residual biomass. Cell viability, reported in Figure 6, showed that both extracts were biocompatible with both the cell lines analyzed, up to 200 µg mL^−1^.

**Figure 7:**
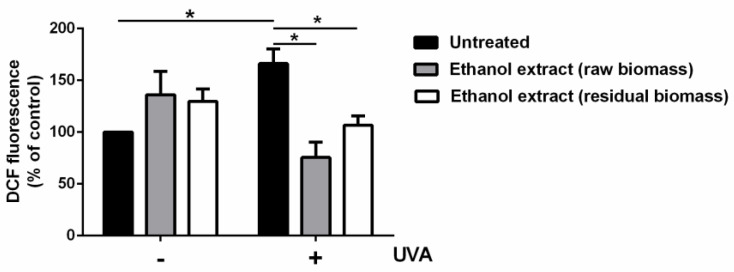
Antioxidant activity of carotenoids from *Synechocystis* sp. PCC6803. Intracellular ROS levels were determined by DCFDA assay. Cells were pre-incubated in the presence of 80 μg mL^−1^ of extract obtained from raw biomass (grey bars) or from residual biomass (white bars) for 2 h, prior to UVA treatment (100 J cm^−2^). Black bars refer to untreated cells in the absence (−) or in the presence (+) of UVA stress. Values are expressed as percentage with respect to control (i.e., untreated) cells. Data are shown as means ± SD (*n* ≥ 3). * indicates *p* < 0.05. The line is used to show which samples have been used to carry out statistical analysis.

Then, carotenoids extracts, obtained from untreated and residual biomass, were evaluated for their antioxidant capacity, both in vitro and on a cell-based model. First, an ABTS assay was performed and the IC_50_ values (the concentration of extract necessary to inhibit 50% of the free radical ABTS^•+^) were determined. The IC_50_ of 0.09 ± 0.01 mg mL^−1^ for the extract obtained from the untreated biomass was similar to that obtained on the residual biomass (0.10 ± 0.01 mg mL^−1^). Based on these results, the antioxidant activity was tested on a well-established cell-based system [29], in which immortalized human keratinocytes (HaCaT) were incubated with the extracts in the presence or absence of UVA, used as a source of oxidative stress. In particular, cells were incubated for 2 h with 80 µg mL^−1^ of each extract prior to UVA exposure (100 J cm^−2^). At the end of the experiment, ROS levels were measured by using 2′,7′-dichlorodihydrofluorescein diacetate (H_2_-DCFDA). As shown in Figure 7, an increase in ROS levels was observed when cells were stressed by UVA lamps (166%), whereas, when cells were pre-incubated with each extract, prior to UVA exposure, no increase in ROS levels was observed (white and grey bars, right side of Figure 7).

#### 2.3.3. Lipidic Fraction

**Figure 8:**
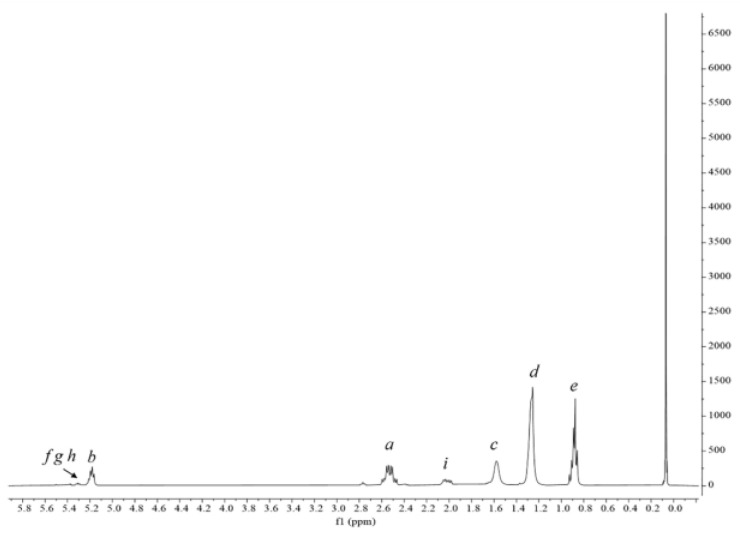
^1^H-NMR spectrum of mcl-PHA.

The lipidic extracts, derived from both the raw biomass and the residual biomass (after protein and pigment extraction) were evaluated for a new purpose, i.e., as carbon sources for polyhydroxyalkanoates (PHA) production by using a well-established bacterial strain: *Pseudomonas resinovorans* [30]. Cells were incubated in the presence of 0.6% *w/v* of each lipidic extract and microbial growth and polymer production were evaluated after 72 h of incubation. No growth was observed in the presence of the lipidic extract derived from the raw biomass, probably due to the toxic effect of co-extracted pigments, phenols and other secondary metabolites, all synergistically acting as antimicrobial agents [31,32]. On the other hand, the microorganism was able to grow in the presence of the lipidic extract derived from the residual biomass, getting up to 1.03 g L^−1^ of cell dry weight and 0.41 g L^−1^ of PHA, that corresponds to 40% w/w of polymer accumulation. H-NMR spectroscopy on neat polymer allowed the confirmation of the presence of signals characterizing medium-chain length (mcl) PHA. In the spectrum (Figure 8), the peaks *a* and *b* are assigned to the protons next to the carboxyl and hydroxy groups, whilst the peak *c* is associated to the first CH_2_ of a saturated alkane side chain. Peaks *d* and *e* are attributed to the remaining CH_2_ groups and the CH_3_ group, respectively. The chemical shifts of peaks *f*, *g*, *h*, and *i* indicate the presence of an unsaturated group in some of the monomers. To the best of our knowledge, this is the first evidence of PHA production starting from lipids obtained by microalgae in a cascade approach. Indeed, only carbohydrates from microalgal hydrolysate have been used as carbon source for the production of PHA through bacterial fermentation [33], or, as an alternative, genetically modified *Synechocystis* has been used as an eco-friendly alternative in the bioplastic production [34]. PHAs represent a high added-value product, with their market expected to reach USD 167 million by 2027, at a CAGR of 15.3% from USD 81 million in 2022 (https://www.marketsandmarkets.com/Market-Reports/pha-market-395.html?gclid=Cj0KCQjwqoibBhDUARIsAH2OpWggbcRYyAWhOURhlonQ1i3du4kI1PQ4gbMm_jopGGoDZH23Kn3-_IcaAo_yEALw_wcB, accessed on 14 February 2023).

## 3. Materials and Methods

### 3.1. Reagents

All solvents, reagents and chemicals, unless differently specified, were purchased from Sigma-Aldrich (St. Louis, MO, USA).

### 3.2. Synechocystis sp. PCC6803 Cultivation

*Synechocystis* sp. PCC6803 was grown autotrophically in bubble column photobioreactors (PBRs) inoculated with BG-11 medium at pH 6.5 and grown at 30 ± 1 °C, equipped with lamps which emitted light at a constant intensity of 200 PAR (μmol_photons_ m^−2^s^−1^). A sterilized gas stream was sparged at the bottom of PBRs using a hydrophobic filter (0.2 µm). The head of the PBRs was equipped with three ports for gas inlet/outlet and sampling operations. The growth was monitored for 14 days by measuring the absorbance at 730 nm, value related to the biomass concentration and indicated by optical density (O.D.).

Division per day (*k*) is directly related to the ratio between the O.D. of each day (*t*_2_) and the initial time (*t*_1_) and inversely related to Δ*t* [35]:

(1) $$k=\frac{{log}_{2}\left({O\text{.}D\text{.}}_{t_{2}}/{O\text{.}D\text{.}}_{t_{1}}\right)}{∆t}$$

### 3.3. Proteins Extraction and Quantification

The biomass was harvested by centrifugation at 1200× *g* for 30 min at r.t. and resuspended in 50 mM sodium acetate buffer pH 5.5. Cells were disrupted as reported by D’Elia et al. [29] with some modifications. Briefly, cells were disrupted by ultrasonicator (Bandelin SONOPULS HD 3200, tip MS73) for 45 min on ice (30 s on and 30 s off) and the extract was then centrifuged at 5000× *g* at 4 °C for 30 min. The recovered aqueous supernatant, after centrifugation, contained soluble proteins. Proteins were quantified by Bradford assay and phycocyanin (PC) concentration was determined spectrophotometrically, according to Bennet and Bogorad equation [36]:

(2) $$C_{PC}\left(mg/mL\right)=\frac{\left[{Abs}_{615nm}-\left(0.474\times {Abs}_{652nm}\right)\right]}{5.34}$$

The residual biomass was stored at −20 °C for further extractions.

### 3.4. Pigments Extraction and Characterization

For pigments extraction and identification, a dry biomass, either raw or after proteins extraction, was used. Extractions were performed using ethanol as solvent, as reported by Aremu, with some modifications [37]. Briefly, for each extraction, 200 mg of freeze-dried biomass was suspended in 4 mL of pure ethanol and disrupted by sonication (40% amplitude, 4 min on ice, Bandelin Sonoplus HD 3200, tip MS73). The final volume was adjusted to 20 mL and the mixture was shaken for 24 h at 250 rpm in a dark room at 4 °C. The mixture was then centrifuged at 12,000× *g* for 10 min and the supernatant was collected, dried under nitrogen stream and then stored at −20 °C. Carotenoids identification and quantification was performed by HPLC-DAD-APCI-QTOF-MS/MS, according to a method previously described [38], with some modifications. Briefly, the analysis of the extracts was carried out in an Agilent 1290 UHPLC system (Ultrahigh Performance Liquid Chromatography) equipped with a diode-array detector (DAD), coupled to Agilent 6540 quadrupole-time-of-flight mass spectrometer (q-TOF MS) equipped with an atmospheric pressure chemical ionization (APCI) source, all from Agilent Technologies (Santa Clara, CA, USA). Extracts were dissolved in pure ethanol at 5 mg mL^−1^ and filtered through 0.45 µm nylon filters before analysis. Analyses were conducted under positive ionization mode using the following parameters: capillary voltage, 3.5 kV; drying temperature, 350 °C; vaporizer temperature, 400 °C; drying gas flow rate, 8 L min^−1^; nebulizer gas pressure, 40 psi; corona current (which sets the discharge amperage for the APCI source), 4000 nA. The mass spectrometer was operated in MS and tandem MS modes for the structural analysis of all compounds. The MS and Auto MS/MS modes were set to acquire *m/z* values ranging between 50–1100 and 50–800, respectively, at a scan rate of 5 spectra per second.

### 3.5. Lipid Extraction

Conventional lipidic extraction was carried out by following the original Bligh and Dyer procedure [27] on 300 mg of both residual freeze-dried biomass (i.e., after protein and pigment extraction) and the raw freeze-dried biomass.

### 3.6. Cell Culture and Biocompatibility Assay

Human immortalized keratinocytes (HaCaT) were from Innoprot (Biscay, Spain) and immortalized murine fibroblasts (BALB/c-3T3), and human epidermoid carcinoma (A431) were from ATCC (Manassas, VA, USA). All cell lines were cultured in 10% foetal bovine serum in Dulbecco’s Modified Eagle’s Medium, in the presence of 1% antibiotics and 2 mM L-glutamine, in a 5% CO_2_ humidified atmosphere at 37 °C. HaCaT and A431 cells were seeded in 96-well plates at a density of 2 × 10^3^ cells/well and BALB/c-3T3 at a density of 3 × 10^3^ cells/well. At 24 h after seeding, increasing concentrations of crude extract (0.01 to 0.4 mg mL^−1^) or ethanolic extract (from 10 to 200 μg mL^−1^) were added to the cells. After 48h incubation, cell viability was measured by the MTT assay, as described by D’Elia et al. [29]. Cell viability was expressed as the percentage of viable cells in the presence of the extract compared to control cells. Control cells are represented by the average obtained by two groups: (i) untreated cells, and (ii) cells supplemented with the highest volume of buffer. Each sample was tested in three independent analyses, each carried out in triplicates.

### 3.7. Carotenoids Antioxidant Activity

The in vitro antioxidant activity of each ethanolic extract was evaluated by the ABTS assay (2,2′-azinobis-[3-ethylbenzthiazoline-6-sulfonic acid]), as previously reported [38]. Briefly, the colorimetric assay is based on the reduction of the ABTS^+^ radical by the antioxidant molecules present in the sample. The radical was obtained by mixing 7 mM ABTS with 2.45 mM of potassium persulfate for 16 h at room temperature in the dark. The mixture was then diluted in deionized water to obtain an absorbance of 0.7 ± 0.02 at 734 nm. Extracts, tested at different concentrations, were allowed to react with ABTS for 7 min in the dark and the absorbance was measured at 734 nm again. Trolox (6-hydroxy-2,5,7,8- tetramethylchromane-2-carboxylic acid) was used as standard to obtain a calibration curve. Results are expressed as IC_50_ (µg/mL), i.e., the amount of extract able to inhibit the ABTS radical. Each extract was analyzed at least three times in triplicate. 

The cell-based antioxidant activity was measured by analyzing intracellular ROS production, as described by Gallucci et al. [39]. Briefly, immediately after UVA irradiation, HaCaT cells were incubated with 2′,7′-dichlorodihydrofluorescein diacetate (H_2_-DCFDA). Fluorescence intensity was measured by a Perkin–Elmer LS50 spectrofluorimeter (525 nm emission wavelength, 488 nm excitation wavelength, 300 nm/min scanning speed, 5 slit width for both excitation and emission). ROS production was expressed as percentage of DCF fluorescence intensity of the sample under test, with respect to the untreated cells. Each value was assessed by three independent experiments, each carried out in triplicates.

### 3.8. Microbial Production of Polyhydroxyalkanoates by Pseudomonas resinovorans

*Pseudomonas resinovorans* NRL B-2649 was provided by NCAURARS-USDA (Microbial Culture Collection, Peoria, IL, USA) and employed for mcl-PHA production. Luria Bertani (LB) medium was used for culture reactivation from the cryopreserved stocks and for inoculum preparation. Polymer production was carried out in Minimal Medium E (MME) [40] complemented with 0.6% (*w/v*) of lipidic fraction extracted from the raw biomass and the residual one (after protein and pigment extraction), as carbon source. Shake-flask fermentations were carried out at 30 °C on an orbital shaking incubator with the agitation rate set at 200 rpm. The cells were harvested after 72 h by centrifugation (5500× *g*, 20 min), washed with a 1:1 aqueous isopropanol solution and thus lyophilized for polymer extraction according to Vastano [40]. The neat polymer was analyzed by ^1^H spectroscopy: samples (0.5 mg) were resuspended in deuterated chloroform (500 μL). The ^1^H-NMR spectra were recorded on Bruker DRX-400 (1H NMR: 400 MHz) in CDCl_3_ (internal standard, for ^1^H: CHCl_3_ at 7.26 ppm).

### 3.9. Statistical Analysis

All the experiments were performed in triplicates. Results are presented as mean of results obtained after three independent experiments (mean ± SD) and compared by one-way ANOVA according to the Bonferroni’s method (post hoc) using Graphpad Prism for Windows, version 6.01.

## 4. Conclusions

In conclusion, a new biorefinery approach has been proposed and new applications for the extracted fractions have been suggested. All the extracted class of molecules are biologically active, and a proof-of-concept for the production of PHAs from the lipidic fraction, extracted as the third class of molecules, has been proved. From a market point of view, the obtainment of these classes of molecules may represent a revenue to allow microalgae exploitation more sustainably from an economic point of view. As an example, natural carotenoids have a market which can vary from 350 to 7500 USD kg^−1^ [41], PC has a market price depending on its purity grade, and can range from about 0.13 USD mg^−1^ when used as a food supplement [42], up to 25 USD mg^−1^ for the analytical grade [43]. Finally, PHAs have a market price ranging between 5–24 USD kg^−1^ [44]. However, a more comprehensive techno-economic analysis of the costs related to upstream and downstream costs is needed to have an objective view of the process. Moreover, a real cost analysis can be reliable only considering the scaling up of the process. The approach here proposed provides an unconventional use of algal products, suggesting a valorization of microalgal biomass.

## Figures and Tables

**Table 1:** Concentration values (μg/g_extract_) for zeaxanthin and β-carotene in the target extracts of *Synechocystis* sp. PCC6803.

Retention Time (min)	Compound	Raw Biomass Extract (µg/g_extract_)	Residual Biomass Extract (µg/g_extract_)
8.057	Zeaxanthin	17,551	10,441
18.651	β-Carotene	216	187
19.800	β-Carotene isomer	64	33

## Supplementary Materials

The following supporting information can be downloaded at: https://www.mdpi.com/article/10.3390/molecules28073144/s1, Figure S1: Protein extraction from *Synechocystis* sp. PCC6803; Figure S2: Carotenoids identification; Table S1: Tentatively identification of compounds from *Synechocystis* PCC6803 by HPLC-DAD-APCI-QTOF analysis.

## Sample Availability

Samples of the compounds are not available from the authors.
