# Supplementary material for: An Alternative Exploitation of Synechocystis sp. PCC6803: A Cascade Approach for the Recovery of High Added-Value Products
Source: Molecules. 2023 Mar 31;28(7):3144. doi: 10.3390/molecules28073144 (PMC10095798; doi:10.3390/molecules28073144)
Supplement: Supplementary file 1 [file molecules-28-03144-s001.zip › molecules-2250872-supplementary2.pdf]

*Supplementary materials*

# An alternative exploitation of *Synechocystis* sp. PCC6803: a cascade approach for the recovery of high added-value products

**Paola Imbimbo [1], Luigi D'Elia [1], Iolanda Corrado [1], Gerardo Alvarez-Rivera [2], Antonio Marzocchella [3], Elena Ibáñez [2], Cinzia Pezzella [1], Filipe Branco dos Santos [4] and Daria Maria Monti [1,*]**

*Correspondence: mdmonti@unina.it (D.M.M.); Tel.: +39 081 679150

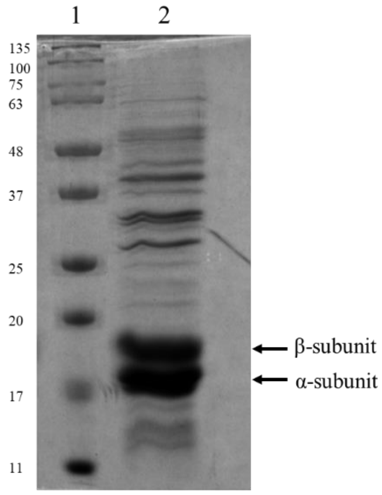


**Figure S1. Protein extraction from *Synechocystis* sp. PCC6803.** SDS-PAGE analysis of protein extract after sonication. Lane 1: molecular weight markers; lane 2: 30 μg of total soluble proteins. After electrophoresis, the gel was stained with Coomassie-Blue Brilliant.

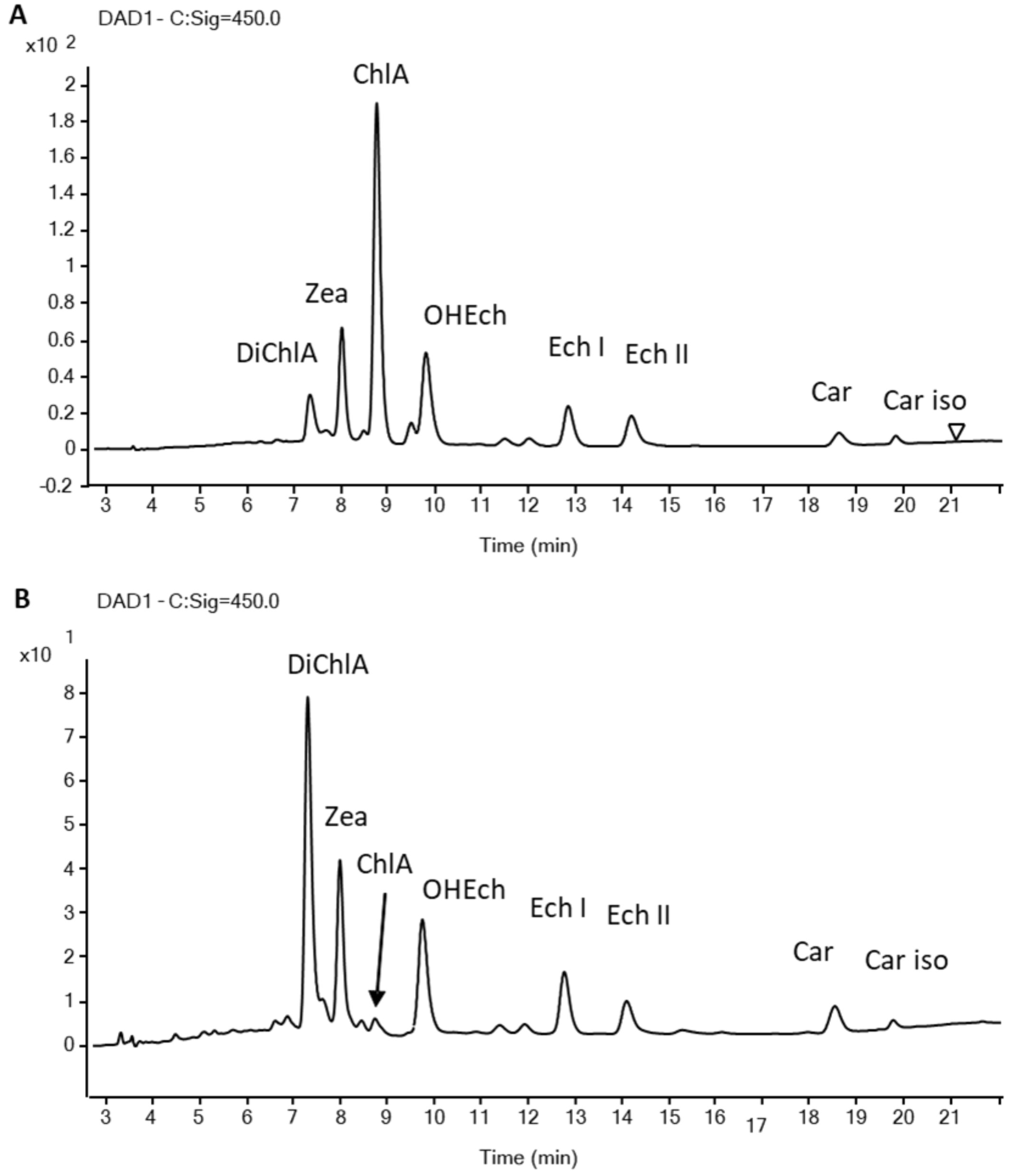


**Figure S2. Carotenoids identification**. Annotated HPLC-DAD chromatograms obtained at 450 nm for the extracts of *Synechocystis* sp. PCC6803 raw biomass (**A**) and *Synechocystis* sp. PCC6803 residual biomass (**B**).

**Table S1.** Tentatively identification of compounds from *Synechocystis* sp. PCC6803 by HPLC-DAD-APCI-QTOF analysis. Peak annotation, high-resolution mass spectrometry features and UV–Vis maxima are reported.

| Peak | RT (min) | Identification | Monoisotopic mass | Theoretical $[M + H]^+$ $m/z$ | Experimental $[M + H]^+$ $m/z$ | Error (ppm) | UV–Vis maxima (nm) |
|---|---|---|---|---|---|---|---|
| DiChlA | 7.419 | Divinylchlorophyll A | 890.5197 | 891.5269 | 891.5260 | -1.06 | 340s, 380s, 428 |
| Zea | 8.057 | Zeaxanthin | 568.4280 | 569.4353 | 569.4346 | -1.24 | 420s, 445, 480 |
| ChlA | 8.778 | Chlorophyll A | 892.5353 | 893.5426 | 893.5450 | 2.69 | 340, 380s,430 |
| OHEch | 9.826 | Hydroxyechinenone | 566.4124 | 567.4197 | 567.4191 | -0.98 | 420s, 445, 476 |
| Ech I | 12.884 | Echinenone I | 550.4175 | 551.4247 | 551.4234 | -2.43 | 460 |
| Ech II | 14.237 | Echinenone II | 550.4175 | 551.4247 | 551.4251 | 0.65 | 460 |
| Caro | 18.650 | β-Carotene | 536.4382 | 537.4455 | 537.4452 | -0.52 | 420s, 450, 480 |
| Car iso | 19.800 | Carotene isomer | 536.4382 | 537.4455 | 537.4440 | -2.75 | 420s, 450, 480 |
